# Social capital and grassroots organisational change: a comparative case study from post‐Morakot Taiwan

**DOI:** 10.1111/disa.70065

**Published:** 2026-06-19

**Authors:** Pak Wan Major Pau

**Affiliations:** ^1^ University of Birmingham United Kingdom

**Keywords:** disaster management, grassroots organisations, recovery, social capital, Taiwan, Typhoon Morakot

## Abstract

Grassroots organisations (GOs) often emerge spontaneously in disaster contexts to fill gaps left by formal authorities, changing their structures and functions to meet evolving community needs. While prior research documents these transformations, it offers limited insight into why they occur and what they mean for disaster recovery. Social capital (SC) is critical for collective action, but its role in shaping grassroots organisational change remains underexplored. This study, examining community‐based GOs in Taiwan following Typhoon Morakot in 2009, investigates how SC influences organisational change. Using a comparative case study design, it finds that bonding SC within communities was most closely associated with whether change was sustained. Alignment between the initiatives of GOs and community priorities mediated bonding SC, sustaining trust, reciprocity, and legitimacy. Bridging and linking SC supported access to resources and external legitimacy, but they were not sufficient on their own when local support weakened. Theoretically, the study clarifies how SC subtypes interact with organisational change and helps interpret what such changes signify. Practically, it underscores the importance of responsive community engagement while strategically leveraging bridging and linking SC in post‐disaster recovery.

## 1 INTRODUCTION AND LITERATURE REVIEW

Historically, much scholarly attention in the field of disaster management has centred on state‐led responses or established humanitarian organisations. Yet, alongside these formal structures, a wide array of actions occur at the grassroots level that remain largely understudied and undertheorised. Individuals (Twigg and Mosel, 2017), local communities (Kitching et al., 2016), volunteer groups (Waldman et al., 2018), faith‐based organisations (Sun and Qi, 2023; Nurdin, 2024), diaspora networks (Hirono, 2024), and self‐organised initiatives (LaLone, 2012; Tu, 2022) often mobilise resources, coordinate assistance, and provide critical support in ways that are distinct from formal authorities. Despite their practical significance, these forms of action are rarely captured by traditional governance frameworks, leaving gaps in our understanding of how and why they emerge, operate, and transform.

The literature uses a variety of terms to describe these phenomena, reflecting overlapping but sometimes divergent conceptualisations. Scholars have referred to them as ‘ad hoc organisations’, ‘emergent actors’, ‘volunteer collectives’, or ‘faith‐based organisations’, among other depictions. Each framing highlights particular aspects—spontaneity, community embeddedness, moral or religious motivation, or informal coordination—but collectively, they point to the existence of organised, localised responses beyond state oversight (Helsloot and Ruitenberg, 2004; Hu, Yeo, and Kapucu, 2022; Krogh and Lo, 2023).

For clarity, this study adopts the inclusive term ‘grassroots organisations’ (GOs) to encompass all of these actors' forms of action, as well as their different types. This framing allows me to focus analytically on the mechanisms and dynamics of grassroots mobilisation without being constrained by terminological differences. Recognising GOs as a distinct category emphasises that disaster response is not solely the domain of formal institutions, but also relies on emergent, localised, and adaptive forms of organisation (Twigg and Mosel, 2017; Rosales, Kilag, and Depoyart, 2023; Das, Becker, and Doyle, 2025). Comprehending GOs is crucial for developing a more complete understanding of disaster management that accounts for the diverse and often transformative nature of organisations operating at the grassroots level.

### 1.1 GOs and typologies

Despite growing recognition of GOs in disaster management, scholars continue to struggle with appreciating their nature. The literature employs a variety of terms—spontaneous, emergent, ad hoc, self‐organised, fluid, and unaffiliated—to describe these entities (Scanlon, Helsloot, and Groenendaal, 2014; Kitching et al., 2016; Twigg and Mosel, 2017; Tu, 2022; Rosales, Kilag, and Depoyart, 2023). Empirical accounts portray GOs as ranging from short‐lived volunteer groups and self‐organised citizen initiatives to faith‐based organisations and diaspora networks (Hirono, 2024; Nurdin, 2024). Some are formally affiliated with institutions, whereas others emerge spontaneously in response to specific events (Wimelius and Strandh, 2024). This heterogeneity highlights that GOs are not a single type of organisation but rather a spectrum of actors, structures, and affiliations, whose composition and form often depend on the local context and disaster‐specific demands.

Other studies focus on what GOs do in practice: mobilise resources; coordinate assistance; and respond to immediate needs that formal authorities cannot always meet (Hu, Yeo, and Kapucu, 2022; Gray, 2023). Their activities may include clearing debris, providing temporary shelter, distributing food and medical supplies, or facilitating communication within affected communities (Tu, 2022). Volunteer engagement may be short‐lived, lasting only a few days (Barraket et al., 2013), whereas faith‐based and diaspora networks often sustain longer‐term involvement through social and cultural ties. Self‐organised initiatives may leverage digital platforms to coordinate localised aid, demonstrating flexibility and adaptability in emergent crises (Liang and Zhong, 2023). Based on these observations, GOs can be conceptualised as bodies of individuals operating in decentralised, localised clusters, responding to immediate and visible needs. Their emergence is situational, contingent on local conditions and gaps in formal responses (Atsumi and Goltz, 2014; Whittaker, McLennan, and Handmer, 2015; Strandh, 2019).

Such diversity points not only to variation in grassroots organisational forms or actions, but also to a deeper theoretical uncertainty about how GOs should be understood as evolving actors in disaster recovery. Not comprehending their nature means that researchers lack a clear explanation of why these organisations emerge, why some dissolve while others persist, and how their roles change over time. More importantly, the meanings and implications of these changes, with respect to grassroots disaster recovery and even longer‐term sustainability, cannot be analysed properly without doing so.

The study of grassroots organisational change is not an entirely blank canvas. Building on the typology of Quarantelli, Dynes, and Haas (1966), Kapucu et al. (2021) distinguished among expanding, extending, and emergent organisations. Expanding organisations maintain their usual tasks at a larger scale or with augmented structures, such as a food bank serving more people because of disaster victims. Extending organisations adopt new tasks while retaining existing structures, such as a church providing temporary shelter without altering its hierarchy. Emergent organisations develop both new structures and tasks, often forming spontaneously to address unmet needs, such as neighbourhood rescue teams during floods. In addition, Carlton, Nissen, and Wong (2022) introduced ‘expectant organisations’, which exhibit all three of the above. The Student Volunteer Army in New Zealand, initially formed for the 2010 Canterbury (New Zealand) earthquake cleanup, expanded and extended its activities, developed temporary structures for volunteer coordination, and repeatedly re‐emerged in subsequent disasters domestically and internationally. Similarly, ad hoc humanitarian groups in refugee crises frequently expand from distributing food to offering health services, psychological support, and legal assistance (Kitching et al., 2016). Local innovation also plays a role. During the early stages of the COVID‐19 (coronavirus disease 2019) pandemic, a Wuhan (China) coffeeshop, Wakanda, provided free coffee to medical staff, before expanding into production and distribution supported by local and distant donors (Gray, 2023). Community‐based recovery initiatives similarly adapt iteratively to shifting needs and available resources (Tu, 2022), blurring the line between expanding and extending forms of grassroots entity. Across cases, GOs transform in response to evolving conditions, whether to increase capacity or meet needs beyond their original mandate.

Despite these insights, existing typologies of grassroots organisational change remain largely descriptive. They categorise structural and functional transformations but do not explain the reason for and the meaning of them. To address this gap, the next subsection turns to the concept of social capital (SC) as a useful lens for interpreting these organisational dynamics.

### 1.2 SC and GOs

SC refers to the network of social relationships that enable actors to access resources, information, and support, often underpinned by trust, norms, and reciprocity (Coleman, 1988; Putnam, 2000). Scholars commonly differentiate three subtypes: bonding SC, which connects homogeneous groups such as families or close‐knit communities; bridging SC, which links heterogeneous groups of similar social status; and linking SC, which spans hierarchical boundaries, connecting grassroots actors with formal institutions (Claridge, 2018; Szreter and Woolcock, 2004). SC is often framed as a mechanism that facilitates cooperation, resource sharing, and trust building.

Within disaster management, SC is frequently cited as a factor enabling collective action, yet often it is unclear how the mechanism of SC leads to collective action. GOs are not pre‐coordinated for crises, so trust and SC become crucial for coordination within and between groups (Park and Park, 2009; Wang, Qi, and Ran, 2022). SC enables members to share resources, information, and responsibilities effectively, even when formal structures are limited (Melo Zurita et al., 2018). Through communication, interaction, and training, SC fosters relational reciprocity and collective trust, enhancing cooperation and mobilisation. In disasters, a high level of SC allows communities to pool resources, coordinate volunteers, and sustain collective action, making it a foundational instrument for rapid response and recovery (Dynes, 2005; Poder, 2011). This study sees GOs as an additional step between SC and collective action. By leveraging the subtypes of SC, namely, bonding, bridging, and linking SC, it has the potential to demonstrate how SC shapes not only the capacity of GOs to act but also the way in which they adapt and sustain efforts in response to disasters.

Notwithstanding this conceptual promise, the existing literature rarely unpacks the mechanisms linking SC to grassroots organisational processes. While studies note that trust, reciprocity, or networks facilitate cooperation, there is limited theoretical or empirical clarity on how different types of SC contribute to the formation and, more importantly, transformation of GOs. This is because GOs are typically not pre‐established entities and must coordinate, adapt, and expand to meet emergent needs. This study addresses this void by examining how SC interacts with grassroots organisational dynamics to produce collective action and, ultimately, more effective disaster recovery, situating SC not merely as a resource but as a structural instrument that underpins the adaptive capacity of GOs.

### 1.3 Research gap and questions

Despite the above insights, a key gap remains in understanding how grassroots organisational change unfolds in a post‐disaster recovery context. Existing studies show that GOs can change structurally and functionally, while SC can facilitate trust, coordination, and resource mobilisation. Yet, the relationship between the two remains insufficiently explained. Consequently, it is still unclear how different forms of SC shape whether GOs adapt, expand, sustain change, fail to transform, or disband over time.

This lacuna is important not only because it leaves the causes of organisational change underexplored, but also because it limits how such changes can be interpreted. GOs do not alter their function or structure for no reason. Without a clearer account of the context‐embedded, social mechanisms involved, it is difficult to assess whether organisational change reflects successful adaptation to community needs, shifting legitimacy and representation, or weakening embeddedness in local networks. In this sense, what is missing is not simply a more comprehensive understanding of the mechanism of grassroots organisational change, but a more relational explanation of how and why such change takes place, and what it means for disaster recovery and the community that a GO represents.

This study asks the following question to address this gap: how does SC affect grassroots organisational change in disaster contexts?

## 2 METHODOLOGY

### 2.1 Research design

This study employs a comparative qualitative design aimed at generating theoretical insights into the relationship between SC and grassroots organisational change in disaster settings. Examples are drawn from communities in Taiwan affected by Typhoon Morakot in 2009, with two villages, Siaolin and Kucapungane, selected for in‐depth, comparative analysis. Within these communities, three GOs serve as primary units of analysis, especially with respect to instances of organisational change within them. Two organisations, the Siaolin Self‐Rescue Organisation and the Taivoan Dance Troupe, are based in Siaolin, while the Rukai Business Development Association (RBDA) operates in Kucapungane.

A comparative case study design is appropriate here given the study's aim of theory building (Yin, 2018). These three GOs represent critical examples (Flyvbjerg, 2006) of where the interaction of SC and grassroots organisational change was particularly visible and consequential. The case study was selected using theoretical sampling, as it offers rich empirical material to test and refine the conceptual framework proposed here. While many communities were affected by Typhoon Morakot, not all of them formed GOs to assist with their disaster recovery effort. Additionally, the three GOs in Kucapungane and Siaolin exhibited different kinds of grassroots organisational change that ultimately reshaped their original structure and functions. This makes the comparison between them ideal for investigating the conditions and mechanisms of SC that enable and prevent grassroots organisational change.

The field survey was conducted in 2023, with two months spent in both Siaolin Village in Kaohsiung City and Kucapungane Village in Pingtung County. Key actors within the selected GOs, as well as relevant external organisations with which they interacted, were identified. To ensure comprehensive coverage and enhance the reliability of the findings, snowball sampling was employed, enabling the identification of additional informants through referrals from initial participants and facilitating a deeper, grounded understanding of local knowledge (Parker, Scott, and Geddes, 2019). Ethical approval for the study was granted by the Humanities and Social Sciences Ethics Committee of the University of Birmingham, United Kingdom (approval number: ERN_23–0488). Informed consent was obtained from all interviewees either verbally (audio‐recorded) or in written signed form.

To capture a holistic view of organisational change during the post‐disaster recovery period from 2009–23, four categories of key informants were identified: three core members of the government's Post‐Disaster Recovery Committee (PDRC); three non‐governmental organisation (NGO) representatives who collaborated with the government; six scholars involved in the recovery process; and eight villagers from each study site (16 in total)—from the Rukai and Taivoan communities—active in the GOs under review (for more information, see Tables A1 and A2 in the Supplementary Materials).^1^ Semi‐structured interviews were conducted with all informants and focused on organisational formation, functional and structural change, internal decision making, community support, and interactions with NGOs and the government during the recovery process.

As well as the observations and interviews, this study also utilised documentary analysis to fill in information gaps as well as to triangulate with existing evidence. First, government documents, such as national and local disaster management policies, local government press releases, legislative resolutions, amendment bills, and minutes of debates were incorporated, including documents of the Executive Yuan, Legislative Yuan, Control Yuan, the PDRC, the Pingtung County Government, and the Kaohsiung Municipality Government. Second, reports from the private sector, NGOs, transnational organisations, and civil society groups were evaluated, such as those by NGOs like the Red Cross Society of Taiwan, Tzuchi, and World Vision, which were major donors, and by the Evergreen Group, which sponsored a school building for children of Kucapungane and others.

A qualitative approach was adopted to explore the relational dynamics of SC and grassroots organisational change among the GOs of the two villages. The analysis draws on transcripts of key informant interviews along with notes from field observations. Given that the study employed a comparative case study design aimed at gaining a deep, contextualised understanding of the issue through direct engagement with communities, qualitative data collection and analysis methods were considered most appropriate (Castellan, 2010). Triangulation was achieved by combining data from the key informant interviews, literature reviews, and evaluations of policy documents/reports, with the findings presented narratively to highlight key themes.

### 2.2 Analytical framework

GOs represent a form of collective action, often emerging to address immediate local needs that formal authorities are unable or unwilling to meet (Hu, Yeo, and Kapucu, 2022; Gray, 2023). The formation and functioning of GOs are strongly supported by SC—the networks, trust, and norms through which communities coordinate collective action (Wang, Qi, and Ran, 2022).

GOs are not static entities, however. As circumstances evolve, they may adjust their functions (such as by shifting from advocacy to economic development) or structures (such as by expanding their membership or creating new roles) (Kapucu et al., 2021; Tu, 2022). While much research has examined the outcomes of such transformations, this framework focuses on how and why GOs change, and the role of SC in shaping the sustainability of those changes.

**FIGURE 1:**
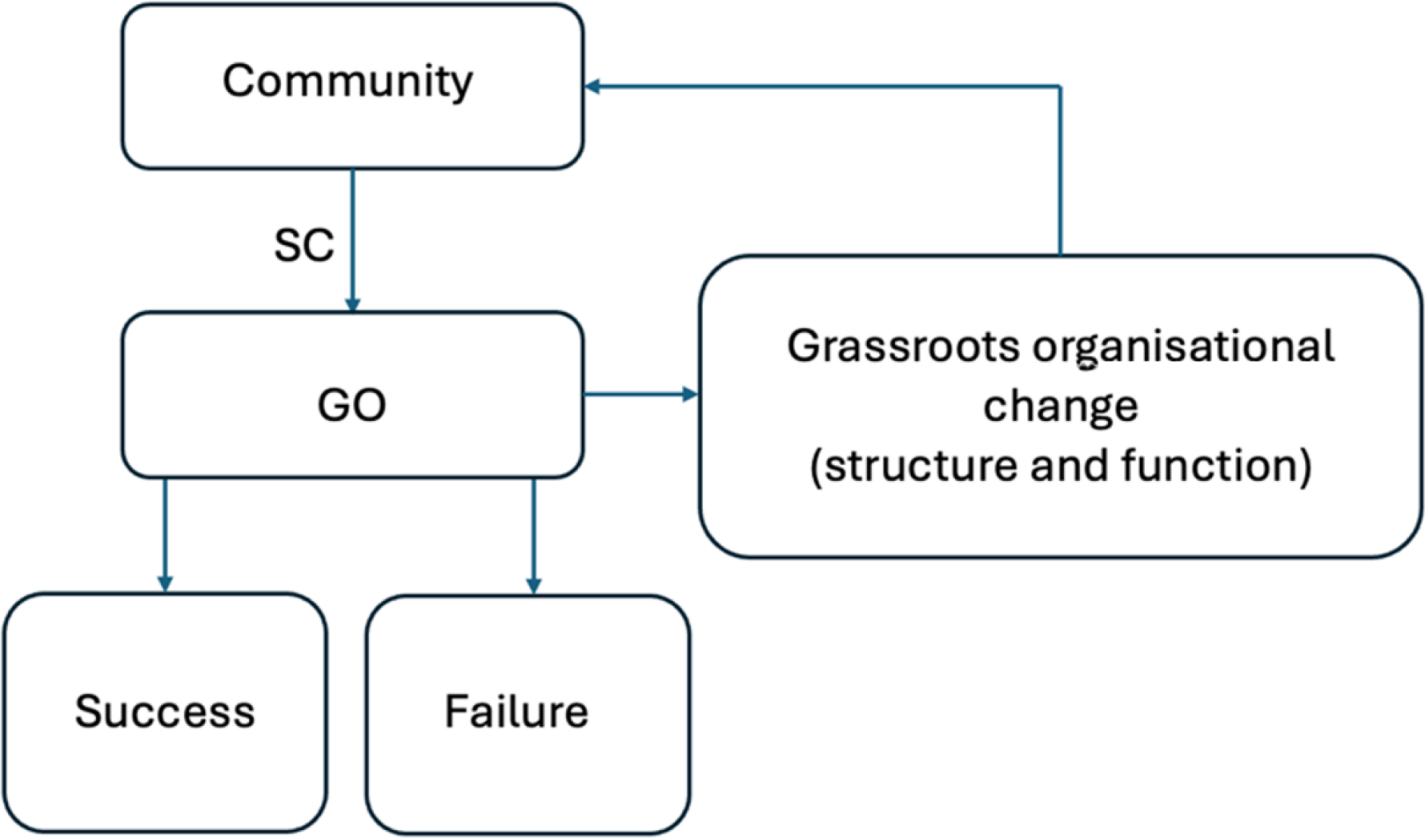
SC and grassroots organisational change. **Source:** author.

SC is particularly relevant because it is voluntary in nature (Lin, 2008). Communities provide or withdraw support depending on whether a GO's changes align with their interests and expectations. A GO may initiate change proactively, yet its sustainability ultimately depends on continued provision of SC by the community. Thus, SC is both a resource and a conditional mechanism: it enables the GO's transformation when community interests are aligned and constrains it when interests diverge. Figure 1 presents the analytical framework of this study.

The proposed analytical framework conceptualises grassroots organisational change as a dynamic process shaped by the interaction between GOs and community support through SC. GOs can independently modify their functions or structures in response to internal reflections, evolving goals, or external pressures. These changes may involve shifting priorities, expanding membership, or developing new initiatives, reflecting the organisation's sensitivity to both internal capacities and environmental conditions.

Once a GO initiates change, the community evaluates whether the new direction aligns with its interests and priorities. SC serves as the key mechanism through which the community's support or withdrawal is communicated. If the change is perceived as beneficial, the community continues to invest its SC, enabling the GO to implement and institutionalise its new functions or structures. Conversely, if the change is not aligned with community expectations, support is withheld, reducing the GO's capacity to sustain its transformation and, in extreme cases, leading to its dissolution. In this framework, SC functions as a conditional enabler: it does not directly cause organisational change but determines whether changes, once initiated, are sustainable. The following subsections reiterate the key concepts of the analytical framework, which underpinned its operationalisation during data collection.

#### 2.2.1 Community

The community is defined as the entire population of the affected village, encompassing both those people directly engaged with GOs and those indirectly affected by their actions. Community interests, preferences, and collective support shape whether GOs can sustain their activities and transformations (Putnam, 2000; Szreter and Woolcock, 2004). The community's response to GOs reflects the voluntary nature of SC and determines whether organisational change aligns with local priorities and continues to receive backing.

#### 2.2.2 Social capital

SC refers to the network of relationships within the community that enable resource sharing, coordination, and collective action. As noted, it is differentiated into bonding SC (strong ties within homogeneous groups), bridging SC (ties across heterogeneous groups), and linking SC (ties connecting grassroots actors to formal institutions) (Coleman, 1988). SC functions as the enabling mechanism that supports the formation, functioning, and potential adaptation of GOs in response to emergent needs (Wang, Qi, and Ran, 2022).

#### 2.2.3 A GO

A GO is a locally embedded, voluntary collective formed to address immediate needs unmet by formal authorities (Twigg and Mosel, 2017). GOs operate in decentralised structures, leveraging SC for mobilisation, coordination, and resource sharing. Their existence is contingent on community recognition and support, which legitimises their actions and ensures continuity. They are not static; rather, they possess the capacity to initiate functional and structural change in response to both internal ambitions and external pressures (Kapucu et al., 2021).

#### 2.2.4 Grassroots organisational change (function/structure)

Grassroots organisational change refers to the modification of a GO's tasks (functional change) or internal arrangements (structural change) in response to evolving circumstances or opportunities (Tu, 2022). Change may be proactive, reflecting strategic adaptation, or reactive, reflecting external pressures or community feedback. Crucially, the sustainability of such change depends on continued community support, as the voluntary nature of SC allows the population to endorse or withdraw backing based on perceived alignment with its collective interests.

#### 2.2.5 Success or failure

The success or failure of a GO is defined strictly by its ability to sustain functional and/or structural change over time. A successful GO maintains sufficient community support, leveraging SC to operationalise new tasks or structures. Conversely, failure occurs when community support is withdrawn, causing the GO to revert to prior forms or even to dissolve (Hu, Yeo, and Kapucu, 2022). This measure captures the interdependence between grassroots initiatives and the community, emphasising that organisational adaptation is meaningful only when socially endorsed.

## 3 FINDINGS

As the focus of this study is on understanding grassroots organisational change, this section looks at how the GOs of Siaolin and Kucapungane Villages implement their structural or functional changes as well as their respective results. Table 1 summarises the organisational changes empirically selected.

**TABLE 1:** Summary of grassroots organisational change among the selected GOs.

			Original purpose	Type of and reason for change	Result
**Siaolin**	Siaolin Self‐Rescue Organisation	First change	Opposing government's housing recovery arrangements	Structural: restructuring for better division of labour to negotiate with the government	Change sustained
		Second change	Housing recovery arrangements secured; negotiation with government concluded	Functional: development of business plan to increase community income	Change failed
	Taivoan Dance Troupe		Self‐initiated collective healing through folk singing and dancing	Structural and functional: collaborate with community organisation to develop tourism	Change sustained
**Kucapungane**	RBDA		Coordinating community effort to develop tourism	Functional: conflict with the government about an unauthorised building	Change failed

**Source:** author.

### 3.1 Siaolin Self‐Rescue Organisation's first change: formation to reorganisation

The Self‐Rescue Organisation in Siaolin formed as a direct result of the community's dispute with the government over post‐disaster housing arrangements. The government proposed relocating villagers to a newly built mega community, where they would cohabit with other communities. The Siaolin villagers, however, collectively advocated for an independent, rebuilt Siaolin Village, reflecting their desire to preserve their way of life and cultural identity. Such disagreement with the government implies that this GO had little linking SC at the outset; however, it enjoyed high levels of bonding SC from its community. Community members contributed substantial resources, labour, and knowledge to support the organisation's goals. Interviewee 7 emphasised this collaborative spirit:

*He [the leader of the Self‐Rescue Organisation] is kind of a natural leader. Right after that speech we felt hopeful for the first time [since the disaster]. He had a pen and a piece of paper and said something like, ‘write down your name and phone number if you want to do this together with me …’. After a few seconds of silence, someone approached the paper and started putting down his name, [and] then one by one, including me, formed a line behind him. … We felt so motivated, it was so touching*.

This dense network of trust and mutual assistance not only facilitated daily coordination but also strengthened the organisation's legitimacy within the village. During the process, it was also supported by a group of scholars who argued the case of the GO with the government by utilising a cultural perspective. Interviewee 10 highlighted their value:

*… with the scholars, the turnout of our community meetings was high … we didn't care how long they take. Because if we didn't care, no one will. … We did everything ourselves: we formulated tactics, we cooked for each other … those who knew patiently explained what was going on to the elders. We were tired and we knew there's still a long way to go, but we kept each other going*.

Even though the negotiation with the government remained difficult, the GO underwent a structural change to operate better. It redefined its internal coordination mechanisms and clarified the division of labour to improve the efficiency of its advocacy efforts. The community supported this adjustment: more than 90 per cent voted in favour during a village meeting.

### 3.2 Siaolin Self‐Rescue Organisation's second change: reorganisation to business development

Following the conclusion of the housing arrangement negotiation with the government, the organisation pursued a second change, a functional one. Its focus was geared towards regenerating the village's livelihood through the development of business. While the goal of economic development aligned broadly with community interests, the proposed business plan failed to resonate with the villagers. It did not reflect local priorities, and as a result, the organisation experienced a withdrawal of bonding SC, as villagers left the GO. Interviewee 9 commented on declining participation:

*If you get resources and you share it with the community … if you take care of the youth and the elderly, I think it is okay. But [this plan] didn't. [The leader of the Self‐Rescue Organisation] did not employ villagers to work with him … he thought the villagers were not obedient enough. He started to bring outsiders in like they were better … he wanted to adopt managerial systems from those big companies. Is it okay? No. We are a community not a company … each community has its own characteristics … we should develop our business model based on our attributes … that's why we left*.

This misalignment between organisational initiative and community preference led to the Self‐Rescue Organisation's disbandment, despite it enjoying at that stage a high level of resources from external bridging and linking SC.

### 3.3 Taivoan Dance Troupe's first change: self‐healing to business collaboration

The Taivoan Dance Troupe emerged shortly after Typhoon Morakot as a form of emotional support and internal self‐healing. Community members, seeking ways to process trauma and rebuild social bonds, voluntarily participated in performances and rehearsals, sharing their skills, time, and energy. The dance troupe provided a space for mutual encouragement, emotional expression, and communal cohesion, reflecting the immediate needs of the village in the aftermath of the disaster. This initial purpose resonated strongly with the community, ensuring robust support and participation. Interviewee 3 remarked:

*When we started dancing, we just wanted to dance together and slowly process our sorrow. … [Now] I really enjoy performing with the rest of the troupe. … I am not good with words, but I am sure you will understand our story once you see us perform. As a group, I never ran out of confidence*.

The dance troupe's functioning relied heavily on bonding SC within the village. Community members contributed resources such as rehearsal spaces, costumes, and materials, and attended performances regularly. Participation was entirely voluntary, highlighting the voluntary and trust‐based nature of SC. This GO's early activities strengthened interpersonal networks within the village and reinforced trust, which became a foundation for future organisational adaptations.

As members became more skilled and confident, the dance troupe experienced its first change. It underwent a structural transformation by collaborating with a community organisation, to develop tourism initiatives in the village. While a merger exists between the leadership of both entities, it was a formalised division of labour: the partner organisation managed catering and produce development, while the Taivoan Dance Troupe focused on performing and entertaining visitors. This structural change was designed to integrate the troupe's performances into the village's broader economic and cultural revitalisation strategy. The community continued to support this collaboration, recognising the alignment of the dance troupe's activities with communal interests and priorities. Interviewee 12 explained:

*We are all parts of the Siaolin Village, and we regained a lot in the past few years … we have our own community … we relearn our culture through our dances … it's time to showcase it to the outside world. … The community worked very hard to make this happen, our tourism and our business … to tell them we have recovered … [we are] back on our feet, and all thanks to their support from the beginning*.

Furthermore, as the collaboration sustained, the Taivoan Dance Troupe gradually attracted more resources externally from the government. For instance, the municipal government even invited it to perform, on behalf of Taiwan, in an international cultural festival.

### 3.4 The RBDA's first change: business coordination to confrontation

The RBDA emerged in response to a growing demand for tourism‐driven economic development in Kucapungane. Its initial purpose was to coordinate community members willing to invest time and resources in tourism enterprises, such as guide services, craft production, and accommodation. The organisation's formation reflected a deliberate mobilisation of committed villagers who shared a common interest in developing tourism as a livelihood strategy. At this early stage, the RBDA focused exclusively on business activities, and its initiatives were generally aligned with the wider community interest. Interviewee 1 pointed out:

*First, it [tourism] brings cash into the community. … [Second,] when we perform to our guests, not only can we introduce, or promote, our culture to outsiders, it also gives us an opportunity to remember our own culture and integrate it into our daily lives. Also [third], in a lot of these houses there are no young people [young adults], as they work in the urban areas. Only their grandparents live here. Through arranging guests in these houses … it gives our elders something to do … a purpose or at least a chance to talk to people*.

As the RBDA sought to expand its business operations, it constructed a new building intended to support larger‐scale tourism activities and satisfy growing operational needs. Up until this point, the organisation's functional and structural changes remained within the scope of its original business‐oriented mission, and the selective support of participating community members was sufficient to maintain its stability.

The government, however, identified the building as non‐compliant with regulations and issued an order for its demolition. The RBDA's leadership made a pivotal decision that signified a fundamental organisational transformation: rather than complying with the government's order, the GO shifted its function from business development to direct opposition, mobilising supporters for protests, sit‐ins, and confrontational engagement with the authorities. This represented a significant departure from its initial focus and an unexpected structural adaptation, as the organisation now sought to engage in collective action beyond business development, and to involve the broader community in a political struggle. Interviewee 1, an RBDA member, explained:

*No one wanted to do this [be confrontational], but what choice do we have? We have exhausted every peaceful means [with the authorities], haven't we? … Can you blame us for being angry? … We had to try everything just to have them take us seriously*.

Interviewee 24, another RBDA member, underlined frustration concerning inability to solve the issue through institutional channels:

*We talked to different people, the county government, members of the legislature, the committee [the PDRC], and the Council of Indigenous Peoples [at the central government level]. … None of them can offer concrete help … after all, we could not change anything*.

This functional change created tension within the organisation. The confrontational strategy did not align with the interests of many original supporters, particularly those who valued stable relations with governmental authorities or had invested in tourism enterprises for economic, rather than political, purposes. Interviewee 11, a member of the community who did not support direct opposition to the building demolition order, stated:

*From what I saw, he [RBDA leadership representative] was taking a lead in doing something illegal, setting a bad example. Especially when he tried to frame it as an act of civil disobedience … a lot of us [community members] did not agree with what he was doing*.

As a result, some founding members withdrew their support from the GO, reducing its bonding SC and weakening its internal cohesion. At the same time, the RBDA received limited backing from the broader community, as many villagers were indifferent or opposed to engaging in conflict with the authorities, as evidenced by the quote above. This combination of misaligned interests and partial community support curtailed the GO's capacity for sustained change, demonstrating how reliance on selective SC can constrain both functional adaptation and organisational resilience. Interviewee 2 emphasised:

*A lot of us stopped attending RBDA meetings as we thought it went too far. It was okay to do business together. … Those who originally were not optimistic about developing [tourism in the community] further accused us of causing trouble … it was too much pressure to continue*.

Given that the RBDA's confrontational approach no longer aligned with the interests of its original supporters or the wider community, the SC that had sustained the organisation was withdrawn. As well as straining its relationship with the government, a number of founding members stopped participating, and some broader community members distanced themselves, signalling a clear loss of legitimacy and trust. With support eroded, in terms of bonding SC from the community, the RBDA was unable to remain in defiance of the government. Consequently, the building was eventually demolished, and the organisation's efforts to expand tourism suffered a significant setback.

## 4 DISCUSSION

This section examines how the success or failure of grassroots organisational change is determined by SC in post‐disaster recovery contexts, with a focus on four instances of organisational change across the recovery efforts of three GOs. The central argument is that in the cases assessed here, whether functional or structural changes were sustained depends most immediately on bonding SC, which is closely tied to whether a GO's actions aligned with community interests. Bridging and linking SC remained important for resource access and external legitimacy, but in these instances, they were not sufficient on their own to maintain change when local support weakened. This framework allows for a systematic comparison of these cases of organisational change to understand the mechanisms through which SC determines success or failure, thereby answering the overarching research question: how does SC affect grassroots organisational change in disaster contexts? Table 2 summarises the results.

**TABLE 2:** SC subtypes and the results of grassroots organisational change.

			SC subtypes			Result: change successful
			Bonding	Bridging	Linking	
**Siaolin**	Siaolin Self‐Rescue Organisation	First change	✓	✓	*✗*	✓
		Second change	*✗*	✓	✓	*✗*
	Taivoan Dance Troupe		✓	*✗*	✓	✓
**Kucapungane**	RBDA		*✗*	*✗*	*✗*	*✗*

**Source:** author.

### 4.1 Siaolin Self‐Rescue Organisation's first organisational change: high bonding and bridging SC and low linking SC

In the wake of Typhoon Morakot, the Siaolin Self‐Rescue Organisation initiated its first major organisational change to coordinate better housing and relief efforts. At that time, it had low linking SC, struggling to negotiate with government authorities. Yet, the change aligned closely with the community's interests, particularly because the organisation successfully resolved housing issues that had persisted for three years.

As a result, the community continued to provide strong bonding SC, allowing organisational change to be successfully implemented and institutionalised, particularly in relation to housing coordination and volunteer mobilisation activities, which became the foundation for subsequent initiatives. Gradually, the Siaolin Self‐Rescue Organisation also activated bridging SC to access complementary resources and knowledge of other local entities.

This case illustrates that the alignment of GO actions with community priorities helped to sustain bonding SC, a finding consistent with insights from the disaster management and SC literature (Dynes, 2005; Murray et al., 2020; Huang et al., 2022). It also confirms that GOs embedded in strong social networks can sustain organisational change even without formal or hierarchical ties (Szreter and Woolcock, 2004).

### 4.2 Siaolin Self‐Rescue Organisation's second organisational change: low bonding SC and high bridging and linking SC

The second organisational change involved developing a business plan for long‐term recovery initiatives. While the organisation enjoyed bridging SC (from external NGOs) and linking SC (from formal authorities), the plan diverged from community interests, prioritising administrative and entrepreneurial goals over residents' immediate needs.

In response, the community withdrew support, such as by no longer attending meetings or volunteering for Siaolin Self‐Rescue Organisation activities. Despite continued engagement by external supporters, the organisation could no longer mobilise community members in action towards an aligned goal. The loss of bonding SC led to the rapid failure of organisational change (Jalil et al., 2021; Wu, 2021).

This instance highlights the importance of bonding SC, as external support or formal linkages alone cannot sustain organisational change when community alignment is absent. Theoretically, this underscores the value of the mechanism proposed by Claridge (2018) and Putnam (2000) whereby bonding SC is maintained through perceptions of mutual benefit and shared interest, not merely formalised network connections. Furthermore, this case also confirms that grassroots coordination relies heavily on trust and reciprocity within localised networks (Melo Zurita et al., 2018). Bridging and linking SC may enhance legitimacy or access to resources, but without the underlying trust of the local community, such forms of SC cannot compensate entirely for lost cohesion.

### 4.3 Taivoan Dance Troupe's first organisational change: high bonding, bridging, and linking SC

The Taivoan Dance Troupe initially formed for the purpose of cultural and emotional healing. Strong bonding SC among members was sufficient to sustain its initial organisational change, despite limited bridging or linking SC. As the dance troupe matured, it initiated structural and functional changes by collaborating with another community organisation to develop tourism initiatives. Community support remained robust, reinforcing bonding SC, while bridging SC facilitated collaboration and linking SC enhanced credibility with authorities.

This case illustrates that strong bonding SC enables both functional and structural organisational change, echoing the work of Wang, Qi, and Ran (2022). Linking and bridging SC function as capacity enhancers rather than as primary drivers of success; without community endorsement, these forms of SC alone would not have sufficed. The dance troupe's experience also highlights that GOs transform in response to community needs and resources (Liang and Zhong, 2023), reinforcing the fact that successful change requires social embeddedness rather than formal planning alone.

### 4.4 RBDA's organisational change: low bonding, bridging, and linking SC

The RBDA's key organisational change involved shifting from coordinating local tourism businesses to challenging government actions pertaining to an unauthorised building. From the outset, the organisation lacked bridging and linking SC, and this confrontational approach diverged from community priorities, as residents preferred stability and incremental tourism development.

The community withdrew bonding SC, and the organisational change failed. This case reinforces the point that misalignment with local priorities undermines bonding SC, and bridging and linking SC may not, on their own, be enough to sustain the transformation. Recent studies also confirm this pattern: when grassroots initiatives ignore local needs, community support diminishes rapidly, even in the presence of formal partnerships (Gray, 2023; Das, Becker, and Doyle, 2025). Functionally, the RBDA case shows that without embeddedness in local social networks, even structurally significant organisational changes are unlikely to succeed.

### 4.5 The role of SC mechanisms in sustaining grassroots organisational change

Across the four cases, the findings suggest that the outcome of whether organisational changes were sustained was closely associated with bonding SC, which in turn was shaped by alignment with community interests. Bridging and linking SC facilitate resource access, legitimacy, and collaboration, but in these cases, they were not sufficient to offset weakened bonding SC. These dynamics occur regardless of whether organisational changes are functional or structural, corroborating research showing that social embeddedness, rather than formal structures, enables sustainability (Whittaker, McLennan, and Handmer, 2015). Communities continuously evaluate the actions of GOs for alignment with their interests, adjusting levels of trust, reciprocity, and participation in response (Wang, Qi, and Ran, 2022). This is consistent with the work of Lin (2005) and Claridge (2018), who emphasise that internal cohesion underpins effective collective action.

Nevertheless, these findings should not be interpreted as suggesting that bridging and linking SC are unimportant in all instances of grassroots organisational change. Rather, in the cases examined here, these forms of SC were not sufficient on their own to sustain organisational change once local support weakened. One possible explanation is that, in small village‐based recovery settings, external ties and resources, formal partnerships, and institutional access cannot easily be converted into lasting collective action unless they remain anchored in community trust and perceived alignment with local priorities (Melo Zurita et al., 2018; Wang, Qi, and Ran, 2022). This may reflect the particular conditions of long‐term post‐disaster recovery in rural Taiwan. Conditions such as grassroots organisational legitimacy depend significantly on continued participation based on shared identity within the community and locally grounded expectations. Furthermore, the social embeddedness of recovery initiatives may matter more than directives from formal structures alone (Whittaker, McLennan, and Handmer, 2015). In other contexts, including larger or more socially heterogeneous communities, and different disaster settings, bridging and linking SC may play a more decisive role (Claridge, 2018).

### 4.6 Implications for disaster recovery theory and practice

Theoretically, this study shows that through an examination of SC subtypes, it is possible to identify not only the mechanisms determining how change occurs, but also the social conditions that enable or constrain it. More importantly, this lens strengthens interpretation of what such transformations mean. To establish what constitutes bonding, bridging, or linking SC in a community, a researcher must situate these relations within their social context. The Siaolin Self‐Rescue Organisation's second change and the Taivoan Dance Troupe's change illustrate this clearly. Both were linked to aspirations for a more self‐reliant village economy, yet one failed and the other succeeded. Viewing through the lens of SC, this contrast shows that villagers did not simply support financial independence. Rather, support was sustained when change remained culturally embedded and resonated with collective identity, as in the Taivoan Dance Troupe case, and weakened when it was perceived as less aligned with community priorities, as in the later business turn of the Siaolin Self‐Rescue Organisation. In this sense, SC has strong explanatory power and provides analytical leverage for understanding GO change at a deeper level.

Practically, the findings suggest that GOs and disaster practitioners should prioritise nurturing bonding SC through sustained community engagement and responsive planning. Building bridging and linking SC remains valuable for accessing resources, partnerships, and institutional support, but in the cases examined here, such ties were not sufficient when local legitimacy weakened. Seeing SC as dynamic rather than static may help practitioners to anticipate difficulties in sustaining initiatives as community needs, expectations, and priorities evolve over time. For governmental institutions, this suggests that engagement with GOs may be more effective when it strengthens, rather than bypasses, locally embedded forms of legitimacy and participation. In this sense, external support is likely to be most effective when it complements community trust and reinforces locally grounded organisational relationships, rather than assuming that formal coordination or resource provision alone can secure long‐term sustainability.

These insights reinforce the idea that GOs operate within embedded social networks, and that their ability to survive and adapt is contingent on trust, reciprocity, and alignment with the communities they serve. Overall, the findings contribute to both theory, by clarifying the mechanism that links SC and organisational change, and practice, by providing a roadmap for sustaining grassroots initiatives in post‐disaster contexts.

## 5 CONCLUSION

This study contributes to the field of inquiry first by moving beyond fragmented and often inconsistent understandings of GOs, which have historically been described as emergent, ad hoc, self‐organised, or spontaneous (Scanlon, Helsloot, and Groenendaal, 2014; Twigg and Mosel, 2017; Rosales, Kilag, and Depoyart, 2023). While previous research primarily describes forms and behaviours of GOs, this paper provides a more holistic perspective by systematically examining their structure and function both before and after instances of organisational change. By mapping what GOs were prior to disaster interventions and how they transformed in response to evolving needs, the research clarifies not only the outcomes of change, whether it was successful or failed, but also the pathways through which these entities emerge and adapt. Analysis of grassroots organisational change thus increases our understanding of GOs in disaster management contexts. It illuminates why these organisations exist, how they operate under crisis conditions, and what enables them to adapt and persist. This establishes the foundation necessary to examine deeper mechanisms, highlighting that understanding change is inseparable from comprehending the identity, function, and embeddedness of GOs in their communities.

Building on this knowledge, the study contributes theoretically by offering a nuanced account of how SC shapes the capacity of GOs to implement and sustain organisational change. It demonstrates that the effectiveness of SC depends on both its subtype and its strategic deployment, going beyond the simplistic assumption that more SC is always better (Park and Park, 2009). The findings emphasise that GOs must prioritise and manage SC subtypes strategically, leveraging local trust while coordinating with external networks to ensure adaptability and resilience.

In addition to understanding why some GOs succeed in implementing change while others fail, recognising the meaning of such transformation also improves how post‐disaster recovery can be assessed and supported. It allows researchers and practitioners to distinguish between changes that deepen community legitimacy and those that reflect weakening embeddedness or shifting local priorities. This insight refines findings in the disaster management field, illustrating that SC is not merely a static resource, but also a relational mechanism whose effect is contingent on alignment with community norms and expectations (Poder, 2011; Kim et al., 2020). In doing so, the paper bridges a key gap in the literature, moving beyond descriptive accounts of GOs to explain the mechanisms that enable organisational change and resilience.

Finally, this research contributes by emphasising the dynamic and context‐dependent nature of SC. Rather than being a fixed attribute, SC evolves through interactions between GOs and their communities, shaping the capacity of these entities to respond, adapt, and sustain organisational initiatives in post‐disaster settings (Nguyen‐Trung, Forbes‐Mewett, and Arunachalam, 2020; Panday et al., 2021). This relational perspective enriches both SC theory and literature on grassroots actors (Aldrich, 2012), providing a more nuanced understanding of how grassroots initiatives thrive and how SC subtypes interact in practice. The findings also speak to the disaster management literature by suggesting that successful disaster interventions require attention not only to material resources and coordination structures, but also to the cultivation and maintenance of local social networks and trust.

Despite these contributions, the study has three key limitations. First, the research design is based on a qualitative comparative analysis of select GOs in specific post‐disaster communities. While this approach yields in‐depth theoretical insight, it may limit generalisability to other cultural, geographical, or organisational settings. Second, data collection relied on retrospective accounts of organisational change and community engagement, which may have been subject to recall bias. Third, the study focuses on SC subtypes as primary determinants of organisational change, potentially underestimating the role of other structural, economic, or political factors that may also influence outcomes.

Future research can address these limitations by examining a broader set of communities and organisations across diverse disaster contexts to test the robustness of the mechanisms identified. Longitudinal studies could provide a more dynamic understanding of how SC, and more importantly, different SC subtypes, evolve over time in response to shifting community needs and external pressures. Additionally, integrating quantitative network analysis into qualitative approaches may generate further insights into how and why different SC configurations interact with organisational structures to shape the success or failure of change.

Overall, this study suggests that the success of grassroots organisational change in disaster recovery is relational, shaped by how different forms of SC are cultivated and managed over time, with bonding SC emerging as especially important in the cases examined here. By revealing the mechanisms through which SC affects organisational change, this research offers both theoretical clarity and practical guidance, advancing the literature on identifying the phenomena of grassroots organisational change, the mechanism of change, and based on the cases under review, the meaning of such change. Furthermore, this paper also provides a conceptual roadmap for practitioners aiming to foster resilient community‐based organisations and to engage more constructively with them.

## CONFLICT OF INTEREST STATEMENT

The author declares no potential conflicts of interest with respect to the research, authorship, and/or publication of this article.

## ETHICAL APPROVAL AND INFORMED CONSENT STATEMENTS

*Consent to participate*.

Written informed consent was obtained from all participants prior to data collection. Ethical approval for this study was granted by the Humanities and Social Sciences Ethics Committee of the University of Birmingham (approval number: ERN_23–0488).

*Consent for publication*.

Not applicable. This manuscript does not contain any individual person's data in any form (including images, videos, or identifying details).

## FUNDING STATEMENT

The author received no financial support for the research, authorship, and/or publication of this article.

## Supporting information


**Appendix 1** Interview protocol

**Appendix 2** Interviewee background

## Data Availability

The data that support the findings of this study are not publicly available owing to confidentiality agreements with the participants and ethical approval conditions. The data may be available from the corresponding author upon reasonable request and subject to additional ethical approval.

## Endnote

^1^
All supplementary materials can be found online in the supporting information section at the end of the article.
