# Supplementary material for: Social capital and grassroots organisational change: a comparative case study from post‐Morakot Taiwan
Source: Disasters. 2026 Jun 19;50(3):e70065. doi: 10.1111/disa.70065 (PMC13282451; doi:10.1111/disa.70065)
Supplement: Supplementary file 1 — Appendix 1 Interview protocol [file DISA-50-e70065-s002.docx]

Appendix 1: Interview protocol

| Interviewee groups | Example questions | Literature themes |
| --- | --- | --- |
| Members of grassroots organizations (GOs) | Can you describe how your grassroots organization was first formed after Typhoon Morakot? What motivated you and others to start it? | Emergence and motivation of grassroots actors; unattended grievances (Twigg & Mosel, 2017) |
|  | What kinds of activities or tasks did your organization carry out after you learned the government’s relocation plan? | Roles of GOs in recovery; GOs tasks (Whittaker *et al*., 2015) |
|  | How did you and your members decide what needed to be done first or what problems to focus on? | Roles of GOs in recovery; bonding SC (Scanlon *et al*., 2014; Aldrich, 2012) |
|  | Who were the key people or groups that helped your organization the most, and how did those relationships form? | Bonding and bridging social capital; resource mobilization (Aldrich, 2012; Poder, 2011; Fraser, 2021) |
|  | What made your organization interact with government officials in the way you did? Did such way change over time? | Linking social capital and state-GO interaction; (Lo & Fan, 2020; Aldrich, 2019) |
|  | How did you address the obstacles or disagreements with the government or NGOs? | Reactivation of linking SC (Lo & Fan, 2020; Huang *et al*., 2022) |
|  | How was internal communication and decision-making carried out, especially when there were internal differences? | Internal bonding SC, trust, and coordination (Coleman, 1990; Murray *et al*., 2020) |
| Government officials | How did the committee collaborate with local governments and NGOs in implementing the housing reconstruction program? | Formal authority recovery coordination (Kapucu & Hu, 2020; Wang & Ran, 2023) |
|  | How were community concerns incorporated into these national policies? | Recovery in governance literature (Mizrahi, Vigoda-Gadot & Cohen, 2021) |
|  | When communities opposed the recovery plans designed by the PDRC, how did your team respond to these challenges? | Conflict and negotiation in recovery governance; unresponsive linking SC (Lo & Fan, 2020; Alvarez & Cardenas, 2019) |
|  | Did the PDRC have mechanisms for long-term follow-up with local governments or communities after housing was completed? | long-term recovery (Aldrich, 2019) |
|  | Looking back, how do you perceive the role of grassroots organizations in complementing or contesting government recovery efforts? | GOs’ role; unattended grievances (Cretney, 2016; Whittaker *et al*., 2015) |
|  | What kind of relationships or interactions did you have with GOs during the recovery? | linking SC at local level (Huang *et al*., 2022; Aldrich, 2012) |
|  | Were there cases where community demands differed from the government’s plan? How were those disagreements managed? | Conflict and negotiation in recovery governance; unresponsive linking SC (Lo & Fan, 2020; Alvarez & Cardenas, 2019) |
|  | How did your office coordinate with NGOs or central government agencies when responding to local recovery needs? | Network governance (Kapucu *et al*., 2021) |
|  | Over time, how did your relationship with these GOs evolve as recovery shifted from housing reconstruction to livelihood rebuilding? | linking SC and recovery governance (Aldrich, 2019; Fraser, 2021) |
| NGO staffs | What role did your organization play in the Morakot recovery process, and how were you involved with the PDRC or local governments? | Bridging and linking SC (Wong & Kohler, 2020; Kapucu & Hu, 2020) |
|  | In your experience, how did NGOs and grassroots organizations interact during the recovery? | Bridging and linking SC (Wong & Kohler, 2020; Kapucu & Hu, 2020) |
|  | Were there cases where NGOs were implementing government policies that were unpopular among communities? How did you handle those situations? | Conflict and negotiation in recovery governance; unresponsive linking SC (Lo & Fan, 2020; Alvarez & Cardenas, 2019) |
|  | In other cases, did your organization ever act as a mediator between communities and government agencies? What strategies did you use? | Conflict and negotiation in recovery governance; unresponsive linking SC (Lo & Fan, 2020; Alvarez & Cardenas, 2019) |
|  | How did you establish trust and cooperation with community members and grassroots organizations? | Trust-building and reciprocity in cross-sectoral collaboration (Putnam, 2000; Murray *et al*., 2020) |
| Scholars | From your research or participation, how would you characterize the role of grassroots organizations in Taiwan’s post-Morakot recovery? | GOs’ role; unattended grievances (Cretney, 2016; Whittaker *et al.*, 2015 |
|  | In your view, how did the GOs you interacted or studied mobilize resources, especially when they were opposing government policies? | Resource mobilization; linking SC (Lo & Fan, 2020; Aldrich, 2019) |
|  | What differences have you observed between cases where grassroots organizations were successful and those where they struggled? | Alignment of SC subtypes (Baycan & Öner, 2023; Fraser, 2021) |
|  | In your opinion, how have power dynamics between the state, NGOs, and local communities influenced the outcomes of recovery policies? | GOs’ role; social capital (Wang & Ran, 2023; Lo & Fan, 2020) |
