# Supplementary material for: Social capital and grassroots organisational change: a comparative case study from post‐Morakot Taiwan
Source: Disasters. 2026 Jun 19;50(3):e70065. doi: 10.1111/disa.70065 (PMC13282451; doi:10.1111/disa.70065)
Supplement: Supplementary file 2 — Appendix 2 Interviewee background [file DISA-50-e70065-s001.docx]

Appendix 2: Interviewee background

| Interviewee | Role | Ethnicity |
| --- | --- | --- |
| 1 | Chairman of PDRC | Chinese |
| 2 | Member of PDRC | Chinese |
| 3 | Member of PDRC | Chinese |
| 4 | Red Cross representative | Chinese |
| 5 | Red Cross representative | Pingpu |
| 6 | World Vision representative | Chinese |
| 7 | Scholar in music | Pingpu |
| 8 | Scholar in anthropology | Chinese |
| 9 | Scholar in anthropology | Chinese |
| 10 | Vocational schoolteacher in bakery | Chinese |
| 11 | Scholar in sociology | Chinese |
| 12 | Scholar in politics | Chinese |
| 13 | Siaolin villager- village chief | Taivoan |
| 14 | Siaolin villager- member of SSRO | Taivoan |
| 15 | Siaolin villager- member of SSRO | Taivoan |
| 16 | Siaolin villager- member of SSRO | Taivoan |
| 17 | Siaolin villager- member of dance troupe | Taivoan |
| 18 | Siaolin villager- member of dance troupe | Taivoan |
| 19 | Siaolin villager- member of dance troupe | Taivoan |
| 20 | Siaolin villager- member of dance troupe | Taivoan |
| 21 | Kucapungane villager- village chief | Rukai |
| 22 | Kucapungane villager- former village chief | Rukai |
| 23 | Kucapungane villager- member of RBDA | Rukai |
| 24 | Kucapungane villager- member of RBDA | Rukai |
| 25 | Kucapungane villager- member of RBDA | Rukai |
| 26 | Kucapungane villager- member of RBDA | Rukai |
| 27 | Kucapungane villager | Rukai |
| 28 | Kucapungane villager | Rukai |
